# Involvement of G6PD5 in ABA response during seed germination and root growth in *Arabidopsis*

**DOI:** 10.1186/s12870-019-1647-8

**Published:** 2019-01-30

**Authors:** Lei Yang, Shengwang Wang, Lili Sun, Mengjiao Ruan, Sufang Li, Rui He, Wenya Zhang, Cuifang Liang, Xiaomin Wang, Yurong Bi

**Affiliations:** 1 Ministry of Education Key Laboratory of Cell Activities and Stress Adaptations, School of Life Sciences, Lanzhou University, Lanzhou, Gansu 730000 People’s Republic of China; 2 State Key Laboratory of Plateau Ecology and Agriculture, Qinghai University, Xining, Qinghai 810016 People’s Republic of China

**Keywords:** Abscisic acid, Germination, Glucose-6-phosphate dehydrogenase 5, NADPH oxidases, Reactive oxygen species, Root system architecture

## Abstract

**Background:**

Glucose-6-phosphate dehydrogenase (G6PDH or G6PD) functions in supply of NADPH, which is required for plant defense responses to stresses. However, whether G6PD functions in the abscisic acid (ABA) signaling pathway remains to be elucidated. In this study, we investigated the involvement of the cytosolic G6PD5 in the ABA signaling pathway in* Arabidopsis*.

**Results:**

We characterized the *Arabidopsis* single null mutant *g6pd5.* Phenotypic analysis showed that the mutant is more sensitive to ABA during seed germination and root growth, whereas *G6PD5*-overexpressing plants are less sensitive to ABA compared to wild type (WT). Furthermore, ABA induces excessive accumulation of reactive oxygen species (ROS) in mutant seeds and seedlings. G6PD5 participates in the reduction of H_2_O_2_ to H_2_O in the ascorbate-glutathione cycle. In addition, we found that *G6PD5* suppressed the expression of *Abscisic Acid Insensitive 5* (*ABI5*), the major ABA signaling component in dormancy control. When *G6PD5* was overexpressed, the ABA signaling pathway was inactivated. Consistently, *G6PD5* negatively modulates ABA-blocked primary root growth in the meristem and elongation zones. Of note, the suppression of root elongation by ABA is triggered by the cell cycle B-type cyclin *CYCB1*.

**Conclusions:**

This study showed that G6PD5 is involved in the ABA-mediated seed germination and root growth by suppressing *ABI5*.

**Electronic supplementary material:**

The online version of this article (10.1186/s12870-019-1647-8) contains supplementary material, which is available to authorized users.

## Background

The oxidative pentose phosphate pathway (OPPP) is the main pathway of NADPH production, which is used for biosynthesis [1–4] and redox balance in plant cells [5, 6]. The main regulatory step of OPPP is catalysed by glucose-6-phosphate dehydrogenase (G6PDH or G6PD). Many experiments have proven that *G6PD* is induced by adverse biotic and abiotic stresses, including salinity, drought and ABA [7–11]. Enhanced G6PD activity is linked to the promotion of plant survival and tolerance [9, 11, 12]. *Arabidopsis* genome-wide analysis indicates the presence of two cytosolic (Cy-G6PD) and four plastidial (Pla-G6PD) isoforms of G6PD [13]. The Cy-G6PD includes G6PD5 and G6PD6. Based on the difference in amino acid sequence, the Pla-G6PD is divided into P1, P2 and P0 type: P1 mainly exists in the chloroplast (G6PD1); P2 mainly exists in plastids and some non-oxygen cells (G6PD2, G6PD3), while P0 is a non-functional gene (G6PD4) [13]. Extensive studies indicate that cytosolic and plastidic G6PD play different roles in plant survival and tolerance [9, 11, 12]. For example, Pla-G6PD is crucial in regulating biochemical responses of heavy metals [14], while Cy-G6PD is involved in aluminum toxicity of soybean under high aluminum concentration [15]. In *Arabidopsis*, G6PD6 constitutes an immune signaling module downstream of pattern recognition receptors, linking protein phosphorylation cascades to metabolic regulation [16].

Many stresses cause water deficit and ion imbalance, which leads to inhibition of essential enzymes, destabilization of cell membranes, decrease in nutrient supply, and overproduction of reactive oxygen species (ROS) [12, 17]. ROS serve as signaling molecules to regulate many biological processes including seed germination and root growth in plants [12, 17–19]. It has been documented that ROS are produced through both enzymatic and non-enzymatic reactions in plants [20, 21]. In *Arabidopsis,* ROS are directly originated from AtrbohD and AtrbohF, two ROS-generating NADPH oxidases, impairing stress inhibition of primary root elongation [18, 22]. Recent studies showed that G6PD plays a primary role in stress responses, favoring ROS-scavenging functions [23]. In fact, during drought stress, plant cells increase their needs for reducing power in order to sustain the antioxidant defense system and counteract ROS accumulation and consequent damages [23, 24].

Abscisic acid (ABA) synthesis is significantly induced by stresses and the ABA signaling has an important function in abiotic stress responses, such as seed maturation and dormancy, stomatal closure, and root growth and developmental regulation [19, 25]. ABA-mediated gene regulation occurs through the conserved ABA-responsive elements (ABREs) in gene promoters [26]. ABREs contain ACGT as the core nucleotide sequence, which acts as a binding site for bZIP transcription factors [2, 26, 27]. In *Arabidopsis*, *Abscisic Acid-Insensitive 5* (*ABI5*), a bZIP transcription factor, plays a vital role in mediating ABA signaling during seed maturation [28]. ABA-enhanced stress tolerance is associated with the induction of ROS scavenging systems [29–32]. Furthermore, recent studies showed that ABA affects the activity and expression of barley plastidial G6PD [2]. ABA mediates drought-induced increase of the cytosolic G6PD activity, and the enhanced cytosolic G6PD activity maintains cellular redox homeostasis by regulating the AsA-GSH cycle in soybean roots [11].

With regard to the cytosolic isoforms, the *Arabidopsis* cy-G6PD mutants produce seeds with higher oil content, suggesting that cy-G6PD is essential for the fatty acid metabolism in developing seeds [11, 13]. Interestingly, when *G6PD6* knockout plants were tested for their stress sensitivity, the germination rate of mutant seeds was significantly reduced under salinity conditions and the root growth was strongly affected by NaCl [12]. However, little is known about the expression and function of *G6PD5*. In this work, we used genetic and molecular approaches to study the function of *G6PD5*. We characterized the function of *G6PD5* in seed germination and root growth. In addition, our results demonstrate that G6PD5 functions antagonistically with ABI5 to maintain the ABA signaling level necessary for seed germination and subsequent seedling establishment. We uncovered a novel interplay between ROS, ABA, and G6PD5.

## Methods

### Plant materials and growth conditions

*Arabidopsis thaliana* Col-0 was used as the wild-type. T-DNA insertion mutants *g6pd5* (CS804669) and *g6pd6* (SALK_016157C) were obtained from the Arabidopsis Biological Resource Center (http://www.arabidopsis.org/). The T-DNA in the *g6pd5* mutant is inserted in the coding region of *At3g2300*, and in the *g6pd6* mutant, T-DNA is inserted in the coding region of *At5g40760*. The *G6PD5* overexpressing plants (*OE#1*, *OE#9*) was obtained by transforming the *G6PD5-*containing constructs into Col-0. The following mutants or transgenic lines were also used in this study: *aba2–1*, *aba2–3*, *abi4–102*. The NADPH oxidase gene single mutants *atrbohD1* (CS9555) and *atrbohF1* (CS9557) and the double mutant *atrbohD1/F1* were obtained from the Arabidopsis Biological Resource Center. *ABI5::GUS* was friendly given by Zuhua He (Chinese Academy of Sciences). Seeds of *abi3* and *abi5–2* were provided in courtesy from Yinggao Liu (Shandong Agricultural University, China). The transgenic line *CYCB1;1::GUS* was kindly provided by Guangqin Guo (Lanzhou University, China). All of them are in the Col-0 background. Seeds were sterilized with 1.5% NaClO for 15 min, washed with sterile water for three times, placed at 4 °C for 3 d. Cold-treated seeds were germinated on the half-strength Murashige and Skoog (1/2 MS) medium (pH 5.8) containing 1% sucrose and 0.8% agar in a growth room at 23 °C under 100–120 μmol photons·m^− 2^·s^− 1^ with a 16 h/8 h light/dark photoperiod.

### Phenotypic analysis

In germination assays, WT, *g6pd5*, *OE#1*, and *OE#9* seeds (approximately 50 seeds for each replicate) were surface-sterilized. The seeds were sown on 1/2 MS medium with or without different concentrations of ABA and then incubated at 23 °C with a 16-h light/8-h dark photoperiod. The number of planted and germinated seeds was recorded 5 d after planting. Radicle emergence of > 1 mm indicated seed germination. Three replicate plates were used for each treatment.

In root elongation measurements, *Arabidopsis* seeds were sown on 1/2 MS medium as above, stratified for 3 d, and then germinated at 23 °C for 5 d. For root elongation measurements, 15 seedlings were used per replicate, and three replicates were made for each treatment. Five-day-old seedlings with roots 1–1.5 cm long were transferred from 1/2 MS agar plates onto a new agar medium supplemented with different concentrations of ABA. Increases in root length were measured after 3 d of treatment [33, 34]. The length of primary roots was measured with the NIH Image software (Image J, version 1.43). β-glucuronidase (GUS)-staining sites were counted using an anatomical microscope.

### Generation of plants with different genotypes

To generate *g6pd5* plants harboring *CYCB1;1::GUS* (*g6pd5*/*CYCB1;1* plants) or *ABI5::GUS* (*g6pd5*/*ABI5* plants), the emasculated flowers of *g6pd5* plants were crossed with pollen from Col-0 plants harboring *CYCB1;1::GUS* (Col/*CYCB1;1* plants) or *ABI5::GUS* (Col/*ABI5* plants). The resulting homozygous *g6pd5*/*CYCB1;1* and *g6pd5*/*ABI5* plants were identified by PCR using *G6PD5*-specific primers (left genomic primer, 5΄-CACCATGGGTTCTGGTCAATGGC-3΄; right genomic primer, 5΄-CAATGTAGGAGGGATCTAAATGTAG-3΄) and a T-DNA left-border primer LBb1. Identification of the *CYCB1;1::GUS* and *ABI5::GUS* background was performed by GUS staining; lines in which all seedlings stained blue were used for the experiments.

### Confocal microscopy

For analysis of fluorescence from the probe propidium iodide (PI), seedling roots were stained with PI (Molecular Probes, Eugene, OR, USA) according to the method described by Mei et al. [35]. Seedlings were incubated in the dark with 10 μg/ml of PI for 5–10 min at 25 °C and then washed three times with ddH_2_O. The roots were then imaged under a confocal microscope (Olympus FV 1000; excitation 488 nm, emission 570–650 nm). The length of the elongation zone and the meristem zone in roots, the epidermal cell size, and the cell number in the root elongation region in WT and mutant seedlings were determined after staining with PI under a confocal microscope.

### Histochemical staining and assay of reactive oxygen species (ROS)

The hydrogen peroxide (H_2_O_2_) accumulation was determined using 2,7-dichlorodihydrofluorescein diacetate (H2DCF, Molecular Probes). Seeds of 2-day-old or roots of 5-day-old seedlings were treated with 20 μM H2DCF for 10–15 min, and the fluorescence intensity was monitored under a fluorescence microscope (Olympus FV 1000, excitation 488 nm and emission 500–550 nm). For the determination of H_2_O_2_ or O_2_^.-^ in roots, histochemical staining in root tips was performed using 0.01% (*w*/*v*) 3,3-diaminobenzidine (DAB, pH 5.0, for H_2_O_2_ detection) or 0.005% (w/v) nitrotetrazolium blue chloride (NBT, pH 7.4, for O_2_^.-^ detection), respectively. In all cases, stained materials were photographed using a digital camera (Canon, PC1146). H_2_O_2_ and O_2_^.-^ content were visually detected according to Wang et al. [15].

### Activity determination of antioxidant enzymes, NADPH oxidase and G6PD

Ten-day-old seedlings were soaked in solutions with or without 10 μM ABA for 12 h. After treatment, the activities of antioxidant enzymes, NADPH oxidase and G6PD were evaluated according to the methods of Liu et al. [36] and Wang et al. [37].

### GUS staining

For GUS staining, seedlings were incubated in GUS staining buffer (1 mM 5-bromo-4-chloro-3-indolyl-β-D-glucuronic acid (X-Gluc), 100 mM sodium phosphate (pH 7.5), 0.5 mM K_3_[Fe (CN)_6_], 0.5 mM K_4_[Fe (CN)_6_], 10 mM EDTA, and 0.1% Triton X-100) for 6–18 h at 37 °C and mounted in solution for microscopy analysis [38]. Quantitative GUS activity assay was performed as described by Nan et al. [33].

### Quantitative real-time PCR analysis

Trizol (TaKaRa) was used for the extraction of total RNA from seedlings. After total RNA was digested with the RNase-free DNase I (Promega) for 45 min at 37 °C, it was used for the cDNA synthesis with PrimeScript II 1st Strand cDNA Synthesis Kit (Takara). The diluted cDNA was used for qRT-PCR analysis with SYBR Premix Ex Taq™ II (Takara). Primer sequences used in the study were shown in Additional file 1: Table S1. The cycle threshold 2^(−ΔΔC(T))^-based method was used for relative quantitation of gene expression. Expression levels were normalized to *Actin 2*.

*Arabidopsis* Genome Initiative locus identifiers for the genes mentioned in this article are as follows: *ACTIN2* (AT3G18780), *G6PD5* (AT3G27300), *G6PD6* (AT5G40760), *AtrbohD* (AT5G47910), *AtrbohF* (AT1G64060), *APX1* (At1G07890), *GR2* (At3G54660), *NCED6* (AT3G24220), *NCED9* (AT1G78390), *CYP707A3* (AT5G45340), *CYP707A4* (AT3G19270), *ABI3* (AT3G24650), *ABI4* (AT2G40220), *ABI5* (AT2G36270), *CYCB1;1* (AT4G37490), *PLT1* (AT3G20840), *PLT2* (AT1G51190).

### Generation of G6PD5-overexpressing (G6PD5-OE) lines

Full-length Arabidopsis *G6PD5* cDNA was obtained using reverse transcription PCR, cloned into the pENTR-TOPO cloning vector (Invitrogen) and sequenced. After the LR reaction, *G6PD5* cDNA was inserted into the pGWB2 vector; this construct was named pGWB2-*G6PD5*. Transformed plants were selected on hygromycin-containing medium. Plants of the second generation after transformation were used for the experiments. The empty pGWB5 vector (the ccdb gene was substituted by a non-sense segment with a termination codon) was also transferred into Col-0 and used as control plants.

### Statistical analysis

Each experiment was repeated at least three times. Data were analyzed by one-way variance analysis (ANOVA, *P* < 0.05), and expressed as mean ± SE.

## Results

### Responsiveness of *g6pd* mutants and *G6PD5*-overexpressing lines to ABA treatment during seed germination and root growth

**Fig. 1:**
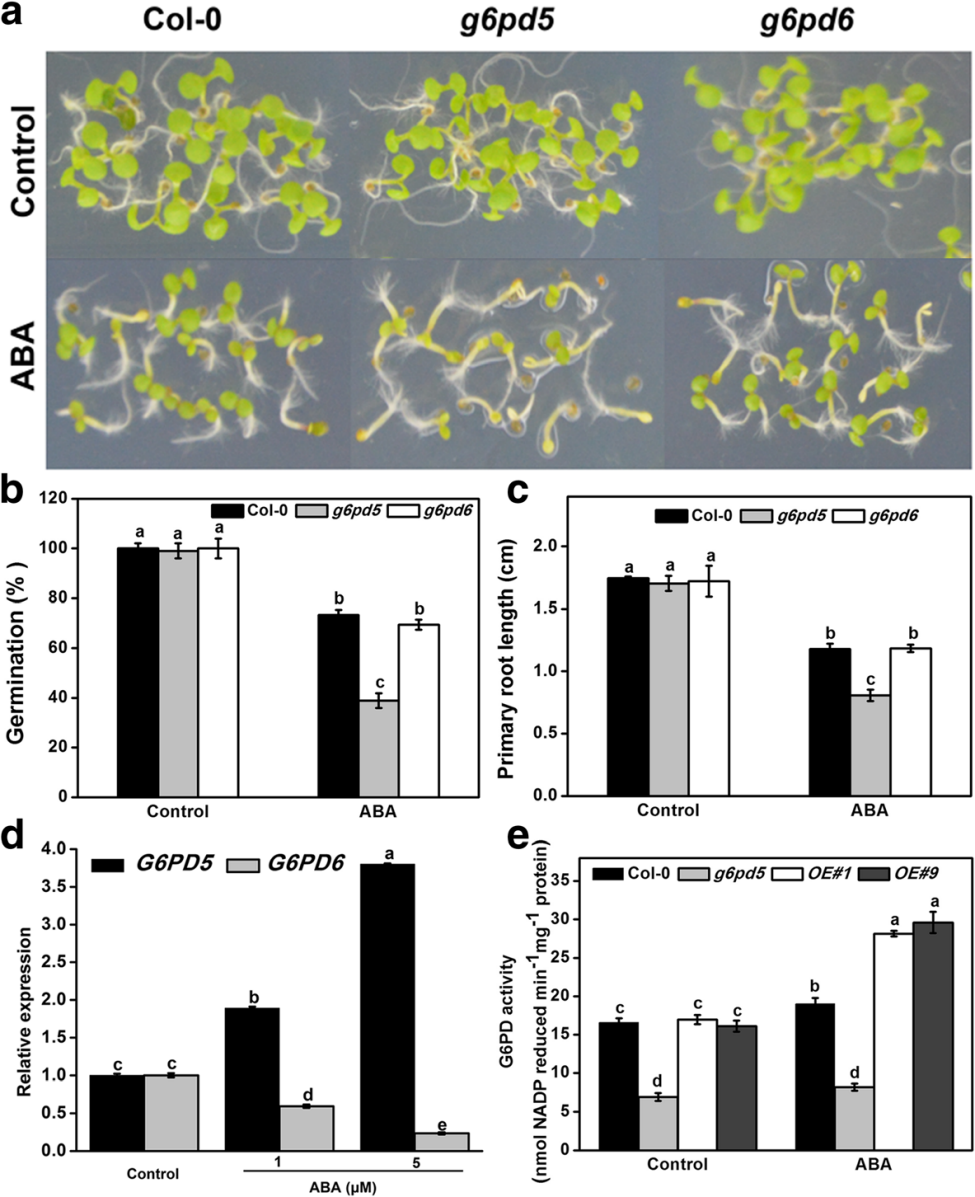
Seed germination and root growth of wild type (WT), *g6pd5*, and *g6pd6* mutant in response to ABA. **a** and **b** Seeds were germinated on 1/2 MS agar plates with 1 μM ABA. Photographs were taken 4 d after treatment. **c** 5-day-old seedlings were grown vertically on 1/2 MS agar plates supplemented with 10 μM ABA. Root growth was monitored and analyzed using ImageJ software. **d** Relative transcript levels of *G6PD5* and *G6PD6* in wild-type (Col-0) with different concentrations of ABA treatment. The transcript levels were normalized to *Actin2*. **e** The activities of G6PD in *Arabidopsis* WT and mutants exposed to ABA treatment. One-way Duncan’s test was performed, and statistically significant differences are indicated by different lower case letters (*P* < 0.05). Bar, 1 cm. The experiments were repeated at least three times with similar results, and data from one representative experiment are presented

**Fig. 2:**
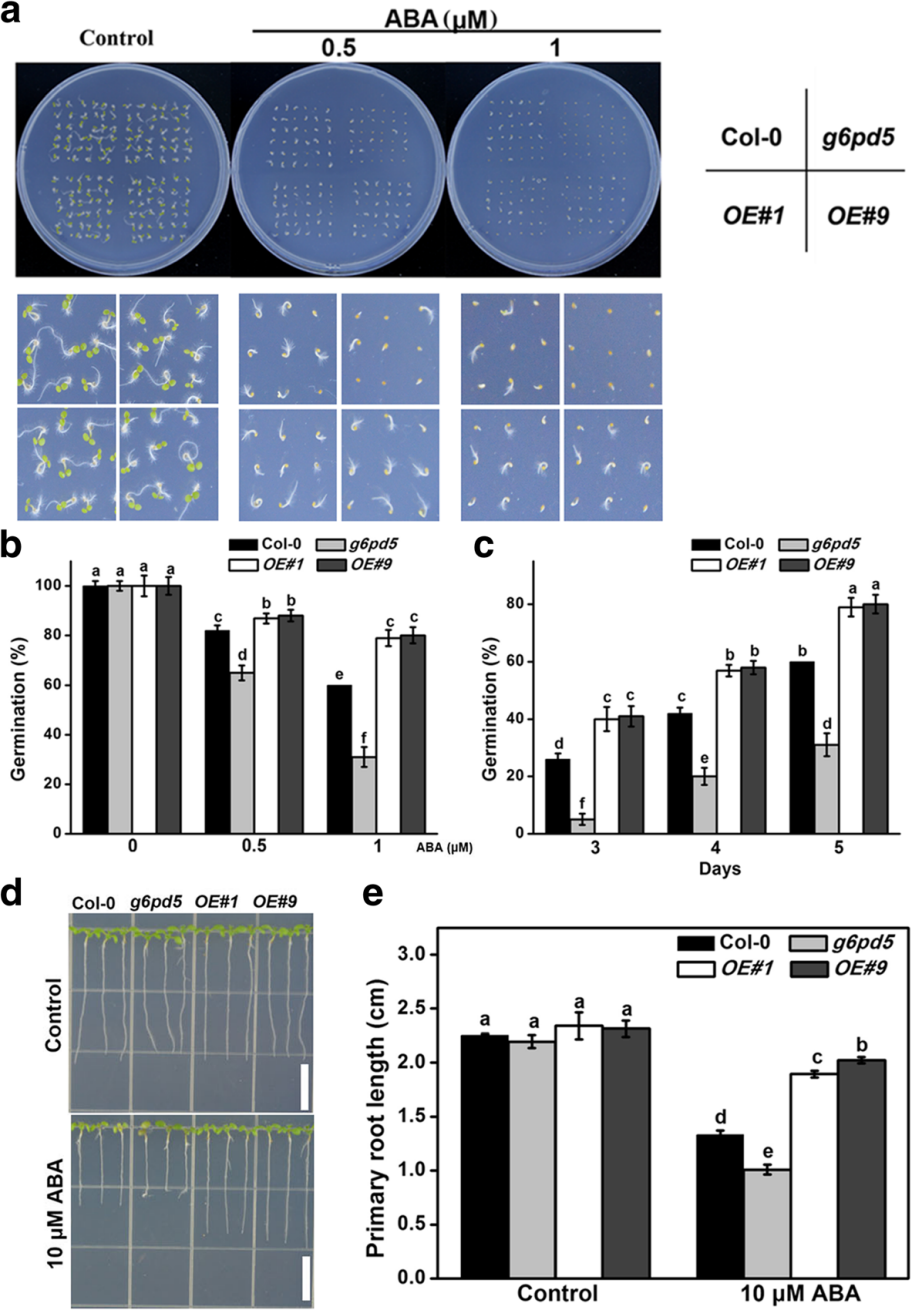
Seed germination and root growth of wild type (WT), *g6pd5* mutant, and *G6PD5-OE* lines in response to ABA. **a** Seeds were germinated on 1/2 MS agar plates with or without ABA. Photographs were taken 4 d after ABA treatment. **b** Percentage of seed germination with or without ABA treatment for 4 d. **c** Percentage of seed germination with 1 μM ABA for 3–5 d. **d** and **e** 5-day-old seedlings were grown vertically on 1/2 MS agar plates supplemented with indicated concentrations of ABA for 3 d and the length of newly grown roots was measured. Root growth was monitored and analyzed using the ImageJ software

ABA is significantly induced by stresses and its signaling has an important function in abiotic stress responses. Therefore, we tested whether G6PD5 is involved in the regulation of ABA signaling. To study the underlying role of G6PD5 in *Arabidopsis*, we obtained a T-DNA insertion mutant from the Arabidopsis Biological Resource Center. The *g6pd5* mutant did not display any visible phenotype at the germination and seedling stages compared to WT under normal growth conditions (Fig. 1a-c). However, the *g6pd5* mutant exhibited severely reduced seed germination rate with increased ABA concentrations compared to WT (Figs. 1, 2a-c). In addition, the primary root growth of *g6pd5* also showed an ABA-sensitive phenotype (Fig. 2d, e). In contrast, seeds of the *g6pd6* mutant exhibited inconspicuous seed germination compared to WT with ABA treatment (Fig. 1a-c). Moreover, the expression of *G6PD5* was induced by ABA treatment, whereas *G6PD6* was inhibited (Fig. 1). Therefore, we mainly focused on *G6PD5* in the following experiments.

Under normal growth conditions, no significant difference in the germination rate and primary root growth was observed between WT and the *G6PD5* overexpression (OE) lines (Fig. 2). However, with ABA treatment, the OE lines exhibited a significantly higher seed germination rate than WT (Fig. 2a, b) and the primary root growth of OE plants was also hyposensitive to ABA treatment (Fig. 2c, d). These data indicate that overexpression of *G6PD5* increases ABA tolerance in *Arabidopsis*.

### Oxidative damage in *G6PD5*-overexpressing and *g6pd5* mutant plants

**Fig. 3:**
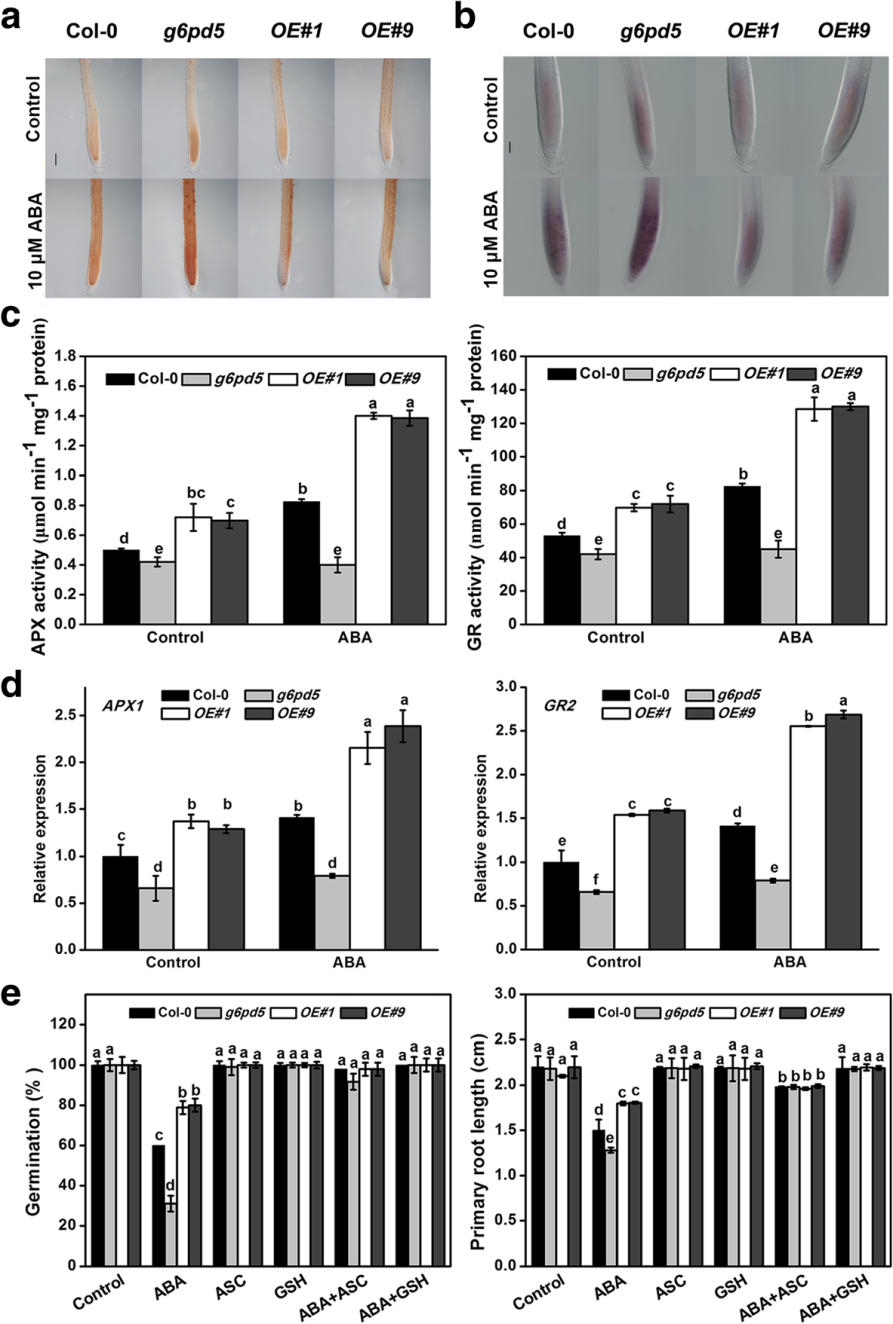
G6PD5 affects the ROS levels with ABA treatment and the activities and transcript levels of antioxidant enzymes in *Arabidopsis* seedlings. 5-day-old seedlings were grown vertically on 1/2 MS agar plates supplemented with the 10 μM ABA for 6 h. **a** Staining of roots with DAB (brown color displaying H_2_O_2_) after ABA treatment. Bar = 100 μm. **b** Staining of roots with NBT (blue color displaying O_2_^.-^) after ABA treatment. Bar = 50 μm. **c** and **d** The activities of antioxidant enzymes and transcript levels of antioxidant enzyme responsive genes. The transcript levels were normalized to *Actin2*. **e** The seeds and seedlings are incubated with 0.25 μM ASC or 5 μM GSH

Previous studies showed that ROS play a key regulatory role in the germination program under abiotic stress [39, 40]. ROS can modulate root elongation by loosening cell walls under favorable conditions [22, 41]. ABA causes oxidative damage and ROS production, so we evaluated the ROS levels in seeds and seedlings of *g6pd5* and G6PD5-overexpressing (OE) lines treated with ABA. The H_2_O_2_ content in both seeds and seedlings of the *g6pd5* mutant and WT increased in response to ABA treatment (Fig. 3a, Additional file 1: Figure S1, Additional file 1: Figure S2a). It is noteworthy that the O_2_^.-^ content was significantly enhanced in the *g6pd5* mutant but attenuated in OE lines (Fig. 3b, Additional file 1: Figure S2b). To further dissect the role of G6PD5 involvement in ROS signaling, exogenous H_2_O_2_ was supplied to the medium. The *g6pd5* mutant showed increased sensitivity to oxidative stress, as manifested by delayed germination and root elongation relative to WT (data not shown). These results suggest that the oxidative level in *g6pd5* is higher than that in WT.

### G6PD5 influences NADPH oxidases AtrbohD and AtrbohF

**Fig. 4:**
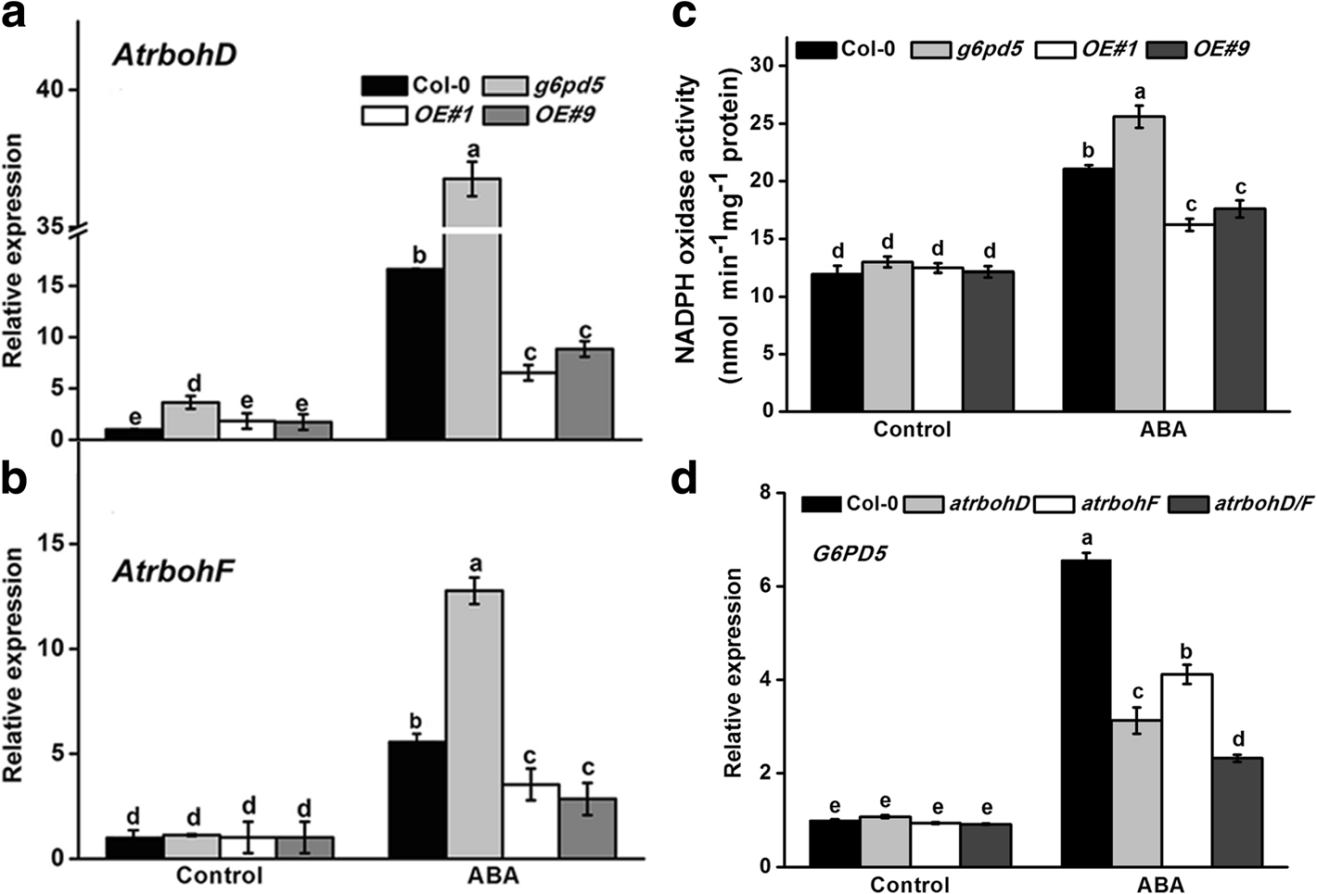
Response of G6PD5 to ABA through NADPH oxidase signaling pathway. **a** and **b** Relative transcript levels of NADPH oxidase genes AtrbohD and AtrbohF in *Arabidopsis* seedlings with or without 20 μM ABA treatment. **c** Activity of the NADPH oxidase in WT and mutants exposed to ABA treatment. **d** Relative transcript levels of *G6PD5* in WT (Col-0) and NADPH oxidase mutant seeds (*atrbohD1*, *atrbohF1*, *atrbohD1/F1*) with or without 20 μM ABA treatment. The transcript levels were normalized to *Actin2*. Results are averages ± SE (*n* = 3). All experiments were repeated at least three times with similar results

In *Arabidopsis*, ROS could be generated by peroxidases, amino oxidases and oxygen photoreduction. However, the main source of ROS production is from the NADPH oxidases AtrbohD and AtrbohF, which play an important role in stress-inhibited primary root growth [22]. To determine whether the function of G6PD5 in response to ABA is achieved through the NADPH oxidase signaling pathway, we analyzed the expression of NADPH oxidase genes in WT and *g6pd5* with or without ABA treatment. As shown in Fig. 4, the expression of the NADPH oxidases *AtrbohD* and *AtrbohF* was markedly increased by ABA treatment in all materials, especially in the *g6pd5* mutant (Fig. 4a, b). These results suggest that *G6PD5* influences the expression of NADPH oxidases. Expectedly, the activity of the NADPH oxidase was higher in *g6pd5* than in WT under ABA treatment (Fig. 4c). These results suggest that cy-G6PD is involved in RBOH-dependent ROS production in ABA-treated seedlings. To prove the hypothesis, we used the NADPH oxidase single mutants, *atrbohD1* (CS9555) and *atrbohF1* (CS9557), and the double mutant *atrbohD1/F1*. In these mutants, the expression of *G6PD5* was lower than that in WT plants (Fig. 4d).

### G6PD5 enhances the expression of antioxidant enzymes

Antioxidant enzymes respond to stresses to remove extra ROS to maintain the equilibrium between ROS production and scavenging [19]. To investigate the effects of G6PD5 on the transcript and activity of antioxidant enzymes, we determined the activities and expression levels of antioxidant enzymes, including *APX* and *GR*. Results showed that the ABA-induced activity and expression levels of *APX* and *GR* (Fig. 3c) in the *g6pd5* mutant were significantly lower than that in WT plants. In contrast, the expression levels of *APX* and *GR* in OE lines were higher than that in WT (Fig. 3d). These results suggest that *G6PD5* enhances the capacity of plants to scavenge excessive ROS under ABA treatment to maintain the balance between ROS production and scavenging. The enhanced G6PD5 activity provides more NADPH for the antioxidant system to remove excess ROS.

Further analysis showed that exogenous application of ascorbic acid (ASC) or glutathione (GSH) partially or fully rescued the seed germination and root growth defects in the *g6pd5* mutant (Fig. 3e). It is noteworthy that GSH was more effective than ASC (Fig. 3f). Therefore, G6PD5 participates in the reduction of H_2_O_2_ to H_2_O in both the glutathione peroxidase cycle and the ascorbate-glutathione cycle.

### G6PD5 affects genes in ABA biosynthesis and catabolism

As a stress phytohormone, ABA has a pivotal regulatory function in numerous stress responses [19, 42]. To determine the function of G6PD5 in ABA metabolism, we analyzed the expression of genes encoding enzymes in ABA biosynthesis and catabolism in WT and *g6pd5* plants. qRT-PCR results showed that ABA biosynthesis genes (*NCED6* and *NCED9*) had higher expression levels in the *g6pd5* mutant than in WT under ABA treatment (Fig. 6a). In contrast, the catabolic genes *CYP707A3* and *CYP707A4* were expressed at an obviously lower level in the *g6pd5* mutant than in WT (Fig. 6a). This result indicates that G6PD5 is involved in the expression regulation of genes in ABA metabolism.

### G6PD5 suppresses the ABA-signaling pathway

**Fig. 5:**
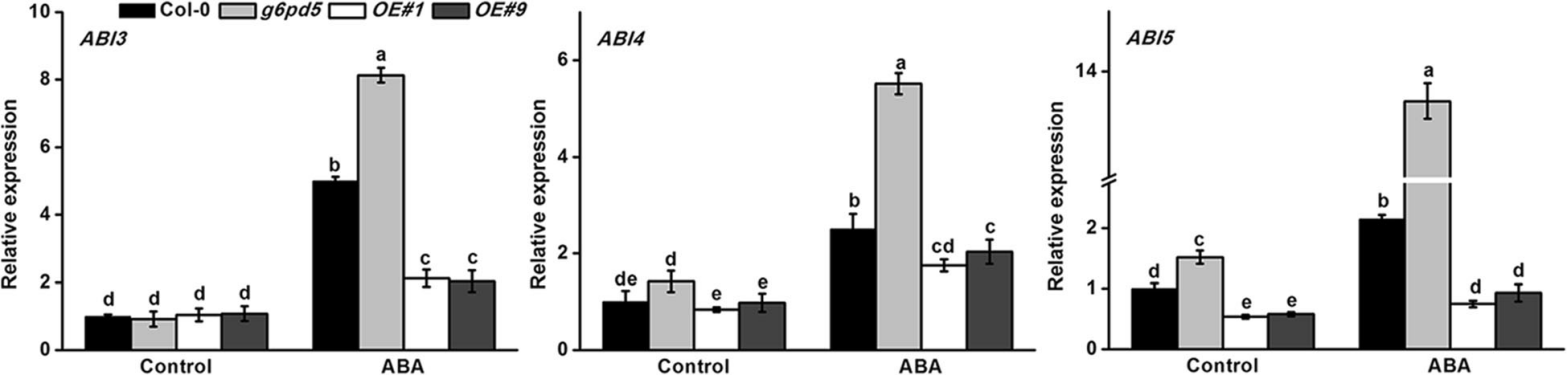
G6PD5 attenuates the expression of several ABA responsive genes. Relative expression levels of ABA-signaling genes *ABI3*, *ABI4*, *ABI5* in plants. Two-week-old seedlings were incubated in liquid MS medium with or without 10 μM ABA for 12 h. The transcript levels were determined by qRT-PCR analysis. The transcript levels were normalized to *Actin2*. Results are averages ± SE (n = 3). All experiments were repeated at least three times with similar results

**Fig. 6:**
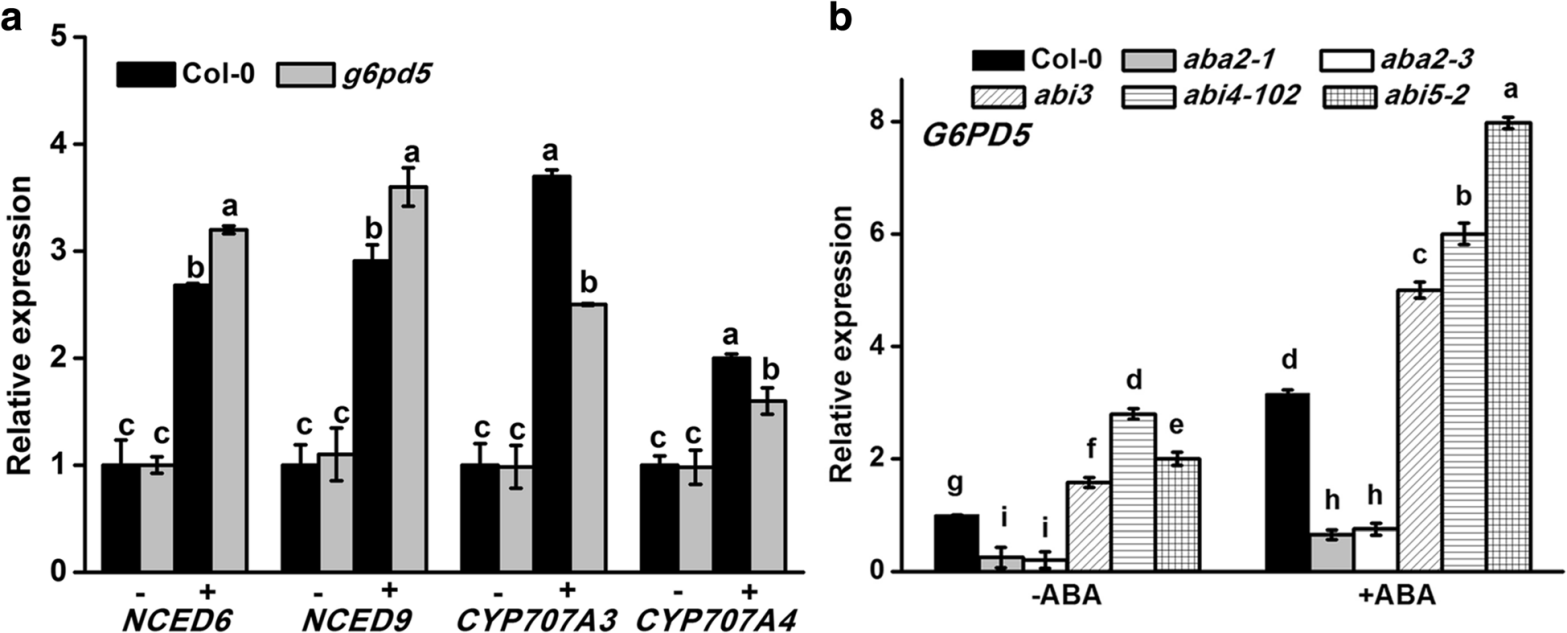
Gene expression. **a** Expression of ABA-biosynthesis genes *NCED6* and *NCED9* and catabolic genes *CYP707A3* and *CYP707A4* in WT and *g6pd5* was analyzed before or after ABA treatment. **b** The expression of *G6PD5* in ABA mutants. Relative transcript levels of *G6PD5* in wild-type (Col-0) and ABA mutant seeds (*aba2–1*, *aba2–3*, *abi4–102*) with or without ABA. In all experiments, the expression of *Actin2* was used as the control. Three replicates were made for each treatment with similar results. Values are mean ± SE of three different experiments. Means denoted by the same letter do not significantly differ at *P* < 0.05 according to Duncan’s multiple range test

ABA signal transduction genes are involved in the regulation of seed germination and root growth [43]. The germination defects in *g6pd5* seeds strongly pointed to the involvement of ABA. We investigated whether differences in the expression of ABA signaling-related genes account for the hypersensitivity of *g6pd5* to ABA (Fig. 5). We examined the expression of ABA signaling genes *ABI3*, *ABI4*, and *ABI5*. qRT-PCR results showed that significant differences in the expression of these ABA-regulated genes were observed between WT and *g6pd5* mutant plants (Fig. 5). The transcript level of *ABI5* was significantly induced in the mutant plant (an increase of 6 fold relative to WT), indicating that *G6PD5* probably acts upstream of these genes in the ABA signaling pathway. We also examined the expression level of *G6PD5* in ABA-deficient mutants (*aba2–1*, *aba2–3*) and ABA-responsive mutant (*abi3*, *abi4–102*, *abi5–2*). Results showed that the *G6PD5* expression level in ABA-deficient mutants (*aba2–1*, *aba2–3*) was markedly lower than that in WT, whereas in ABA-responsive mutants the expression level of *G6PD5* was significantly higher than that in WT (Fig. 6b).

**Fig. 7:**
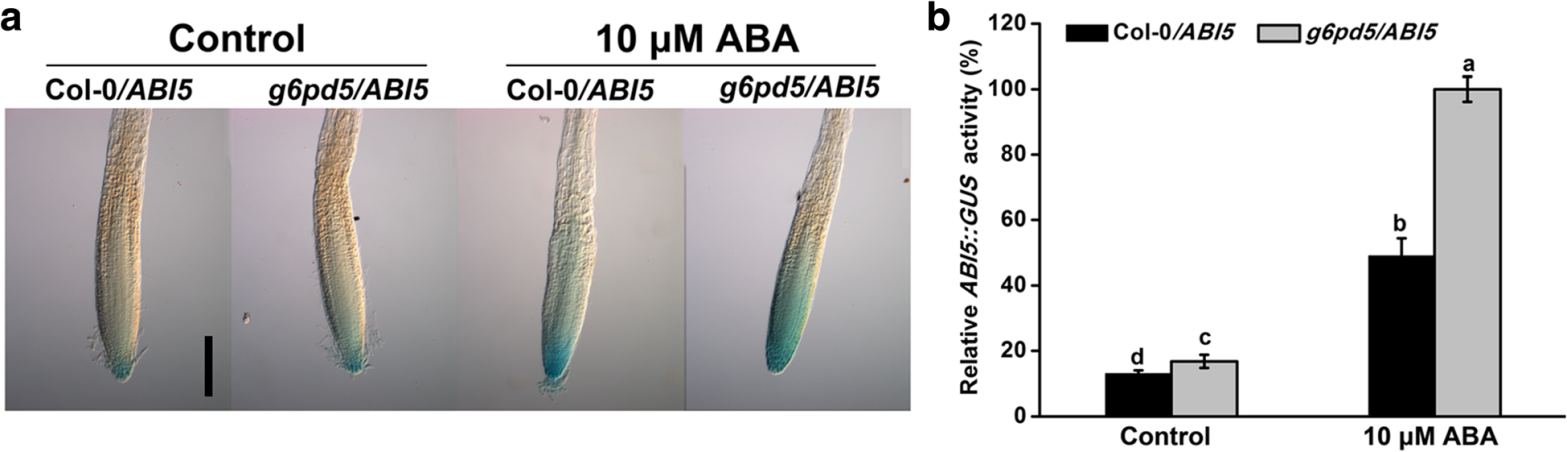
G6PD5 knockout on the expression of *ABI5* genes in *Arabidopsis*. **a** Expression of *ABI5::GUS* in Col-0/*ABI5* and *g6pd5*/*ABI5* seedling roots grown for 7 d then treated with or without 20 μM ABA for 12 h using GUS staining. **b** Quantification of the GUS activity in Col-0/*ABI5* and *g6pd5*/*ABI5* seedlings. The GUS activity of Col-0/*ABI5* root was adjusted to 100%. Mean values and SE were calculated from three independent experiments (*n* = 20). Within each set of experiments, bars with different letters were significantly different at the *p* < 0.05 level

To confirm the *ABI5* expression level in the *g6pd5* mutant, we crossed the *g6pd5* mutant with Col-0 plants harboring *ABI5::GUS* (Col/*ABI5* plants). We obtained homozygous *g6pd5* plants harboring *ABI5::GUS* (*g6pd5*/*ABI5* plants) and compared the *ABI5* expression in Col/*ABI5* and *g6pd5*/*ABI5* seedlings (Fig. 7). Five-day-old *g6pd5*/*ABI5* seedlings showed a strong blue coloration in the QC of root tips relative to Col/*ABI5* with or without ABA treatment (Fig. 7). These results confirmed a higher *ABI5* expression level in *g6pd5* seedlings, suggesting that G6PD5 is likely involved in the regulation of ABA signaling.

### G6PD5 is involved in ABA-inhibited cell elongation in root meristem and elongation zones

**Fig. 8:**
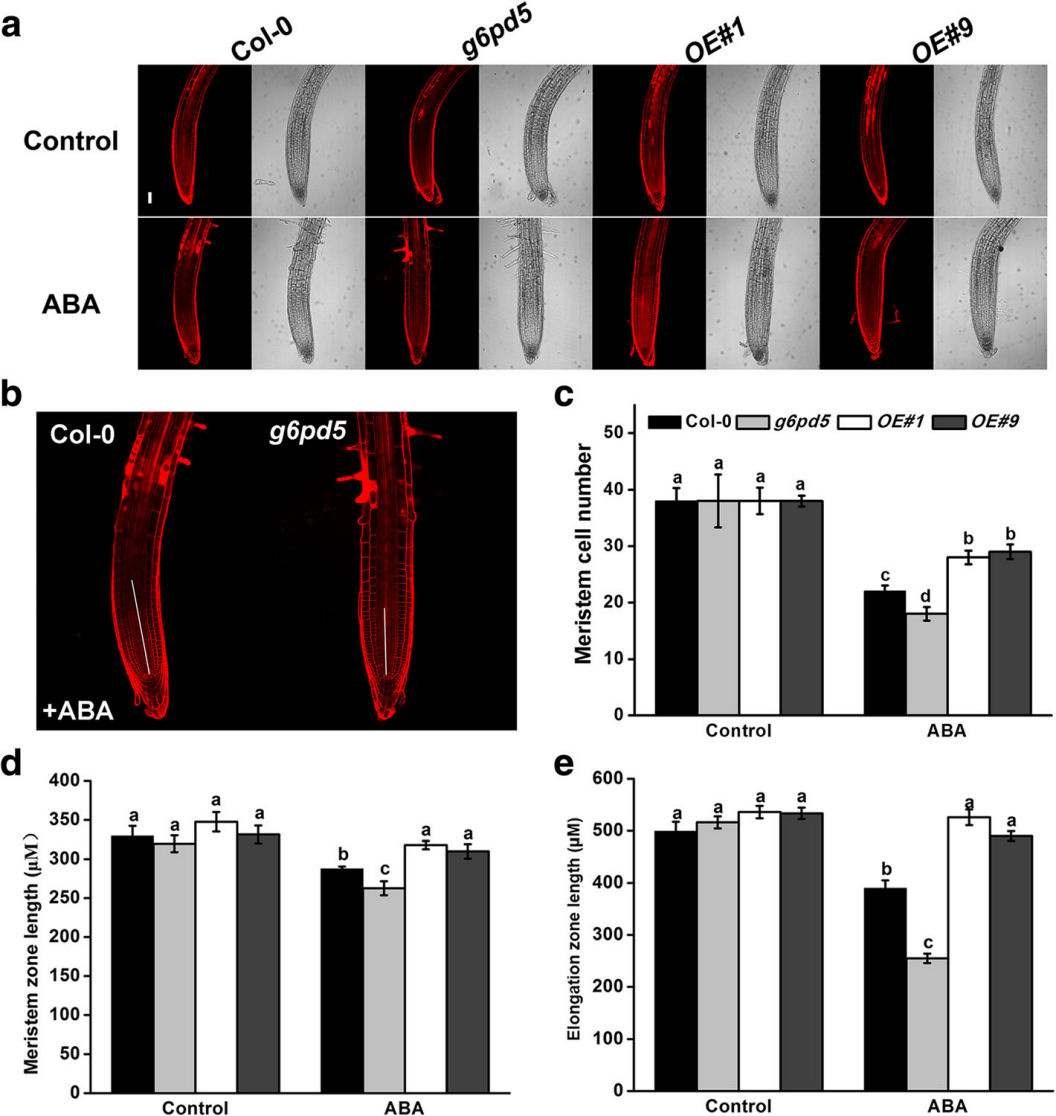
*G6PD5* regulates root meristem and elongation zone. **a** and **b** Root meristems of propidium iodide (PI)-stained images in *Arabidopsis* WT seedlings. Bars = 100 μm. **c** Meristem cell number. **d** Root meristem zone size. **e** Root elongation zone size. The 5-day-old seedlings were treated with 10 μM ABA for 12 h. Except in **a** and **b**, mean values and SE were calculated from three independent experiments (n = 20). Within each set of experiments, bars with different letters were significantly different at the *p* < 0.05 level

To further dissect the mechanisms of G6PD5 function in ABA-repressed root growth in *Arabidopsis*, we measured the primary root length in WT and *g6pd5* mutants grown on 1/2 MS media supplied with ABA. The root growth of WT and mutants was similar on ABA-free medium (Fig. 8a). However, when grown on ABA-containing medium for 12 h, *g6pd5* mutant plants exhibited dramatically shortened root elongation zone compared to WT (Fig. 8a, e). These results indicate that ABA suppresses cell elongation in the elongation zone of *g6pd5* roots. In addition to cell elongation in the elongation zone, cell division in the root meristem zone also contributes to root growth. Therefore, we examined the size of root apical meristem. The length and cell number of the meristem zone in *g6pd* mutant plants were less than that in WT in the presence of ABA (Fig. 8a-d), implying that G6PD5 is required for cell division in the root meristem.

**Fig. 9:**
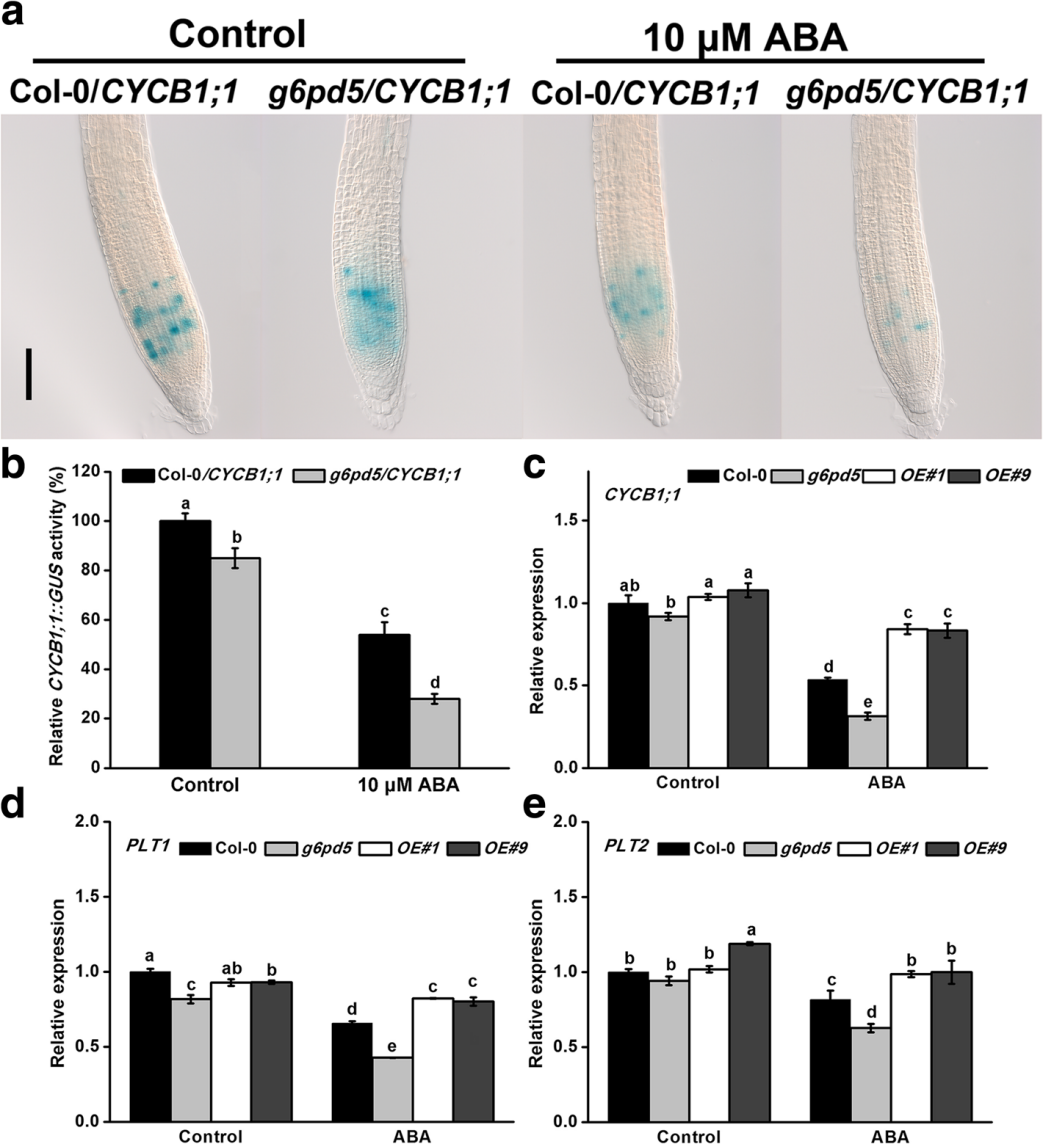
G6PD5 is involved in cell division in the absence or presence of ABA. **a** Expression of *CYCB1;1::GUS* in Col-0/*CYCB1;1* and *g6pd5*/*CYCB1;1* seedlings grown for 5 d with or without ABA treatment for 12 h. Scale bars, 200 μm. **b** Quantification of the GUS activity in *CYCB1;1::GUS* seedlings. The GUS activity in Col-0/*CYCB1;1* roots was adjusted to 100%. **c-e** Relative transcript levels of *CYCB1, PLT1 and PLT2* in WT (Col-0), *g6pd5* mutant and *OE* lines plants with or without ABA treatment. Data are presented as mean values ± SD of three independent experiments. One-way Duncan’s test was performed, and statistically significant differences are indicated by different lower case letters (*P* < 0.05). The experiments were repeated at least three times with similar results, and data from one representative experiment are presented

Furthermore, we found that the cell cycle B-type cyclin *CYCB1* accounts for the reduction in primary root growth in *g6pd5* mutants under ABA treatment (Fig. 9). To further investigate the protective mechanism of G6PD5 under ABA condition, we first examined the expression of *CYCB1* gene in the *g6pd5* mutant and WT. Results showed that the expression of *CYCB1* was decreased under ABA treatment in seedlings, especially in *g6pd5* mutant (Fig. 9c). Next, we used the cell cycle marker line *CYCB1;1::GUS* and observed that homozygous *g6pd5* plants harboring *CYCB1;1::GUS* (*g6pd5*/*CYCB1;1* plants) had weaker blue coloration than Col-0/*CYCB1;1* in the root meristem under ABA treatment (Fig. 9a, b). Based on the above results, we concluded that G6PD5 is involved in the transcriptional regulation of the *CYCB1* gene, and in the process, G6PD5 plays a key role by participating in CYCB accumulation under ABA treatment in primary roots.

The activity of the root meristem is regulated by root meristem-specific genes, among which the PLETHORA (PLTs) genes are master regulators of root development [44]. PLT1 and PLT2 are predominantly localized in the QC and redundantly regulate the root stem cell niche in root apical meristem (RAM) [44]. We investigate the expression of RAM-related genes in WT and *g6pd5*. Results showed that the expression levels of *PLT1* and *PLT2* were lowered in the *g6pd5* mutant compared to WT with ABA treatment (Fig. 9d, e). These findings indicate that G6PD5 is crucial for RAM maintenance in the presence of ABA.

## Discussion

To date, several studies have reported the expression patterns of *G6PDs* during plant development and in response to stresses [11, 37]. Cy-G6PD acts as the main isoforms in the regulatory step of OPPP and is involved in regulating redox balance in cells during salt tolerance [11, 37]. Therefore, Cy-G6PD plays positive roles in enhancing plant tolerance to environmental stresses in several species [23, 45]. In this study, we investigated the involvement of G6PD5 in the response of *Arabidopsis* to ABA during seed germination and root development. We showed that G6PD5 is a central component in seed germination and seedling development under ABA treatment. Seed germination rate of the *g6pd5* mutant under ABA treatment was reduced by approximately 50% compared to WT, implying that G6PD5 has critical functions in plant development and ABA responses (Fig. 2a, b). In contrast, *g6pd6* mutant seeds exhibited inconspicuous germination compared to WT under ABA treatment (Fig. 1a-c). These results suggest that *G6PD5* plays a more important role than *G6PD6* in *Arabidopsis* seed germination. The *g6pd5* mutant exhibited severely reduced seed germination rate and shortened primary roots with increased ABA concentrations compared to WT. Why is *G6PD5* responsive to ABA treatment? We found ABREs in the promoter region of *G6PD5* but not in *G6PD6* (Additional file 1: Table S2).

Under favorable conditions, ROS, such as H_2_O_2_, superoxide anion and hydroxyl radical, are produced at low concentrations in plasma membrane, chloroplasts, mitochondria and peroxisomes [46]. ROS are not only the ever-present danger due to their physicochemical toxicity, but also important signaling molecules that accumulate under many stress conditions [11, 19]. Previous reports showed that H_2_O_2_ plays a role in drought-induced increase of total G6PD activity [11]. Thus, we examined the effect of H_2_O_2_ on *G6PD5* under ABA treatment. We confirmed that the oxidative level is higher in *g6pd5* than in WT. ROS originated from the NADPH oxidases AtrbohD and AtrbohF play an important role in stress-inhibited primary root growth in *Arabidopsis* [22]. Further investigation demonstrated that the ABA-induced H_2_O_2_ generation results from the enhanced NADPH oxidase activity. To consolidate this observation, we examined the antioxidant enzyme activities and transcription levels in *g6pd5* and WT plants (Fig. 3c, d). These results suggest that *G6PD5* is enhanced to scavenge the excessive ROS under ABA treatment in order to maintain the balance of ROS production and scavenging. Results also indicate that the enhanced G6PD5 activity provides more NADPH for the antioxidant system to remove excessive ROS. Reduction of H_2_O_2_ to H_2_O can then be achieved through either the glutathione peroxidase cycle or the ascorbate-glutathione cycle.

ABA plays a central role in plant development and adaptation to numerous stress responses [19, 47, 48]. *ABI3* has long been recognized as the major regulator of seed dormancy and ABA inhibition of seed germination [39, 49]. *ABI5* functions downstream of *ABI3* and promotes dormancy in concert with *ABI3* [39, 43, 50]. Furthermore*, ABI5* plays a role in regulating ROS homeostasis by activating *CATALASE 1* transcription during seed germination [28]. *CATALASE 1* is a key positive regulator of ABA signaling involved in mediating seed germination and subsequent primary root establishment [51]. Mechanistic investigations revealed other important modulators that interact with ABI5 to fine-tune seed germination [51]. *ABI4* is involved in redox regulation and oxidative challenges in *Arabidopsis* leaves [39, 43]. Interestingly, the transcript levels of *ABI3*, *ABI4*, and *ABI5* were increased in the *g6pd5* mutant (Fig. 5). This indicates that G6PD5 probably acts upstream of these genes in the ABA signaling pathway. The *g6pd5* mutant showed less sensitivity to ABA compared with WT. Taken together, our results suggest that the germination defects in the *g6pd5* mutant is mediated by *ABI* genes, especially by *ABI5*.

In addition, ABA inhibition of primary root growth is mediated by the ABA-induced regulation of *CYCB1* expression at the G2/M checkpoint [22, 52]. One mechanism in plant response to primary root growth is to regulate the expression of cell cycle-related genes. We found that the expression of *CYCB1* was decreased under ABA treatment in *g6pd5* mutant seedlings (Fig. 9). Therefore, we propose that G6PD5 is involved in the transcriptional regulation of *CYCB1* gene under ABA treatment. Certainly, G6PD5 is crucial for RAM maintenance in the presence of ABA by regulating the expression levels of *PLT1* and *PLT2* (Fig. 9d, e).

## Conclusions

**Fig. 10:**
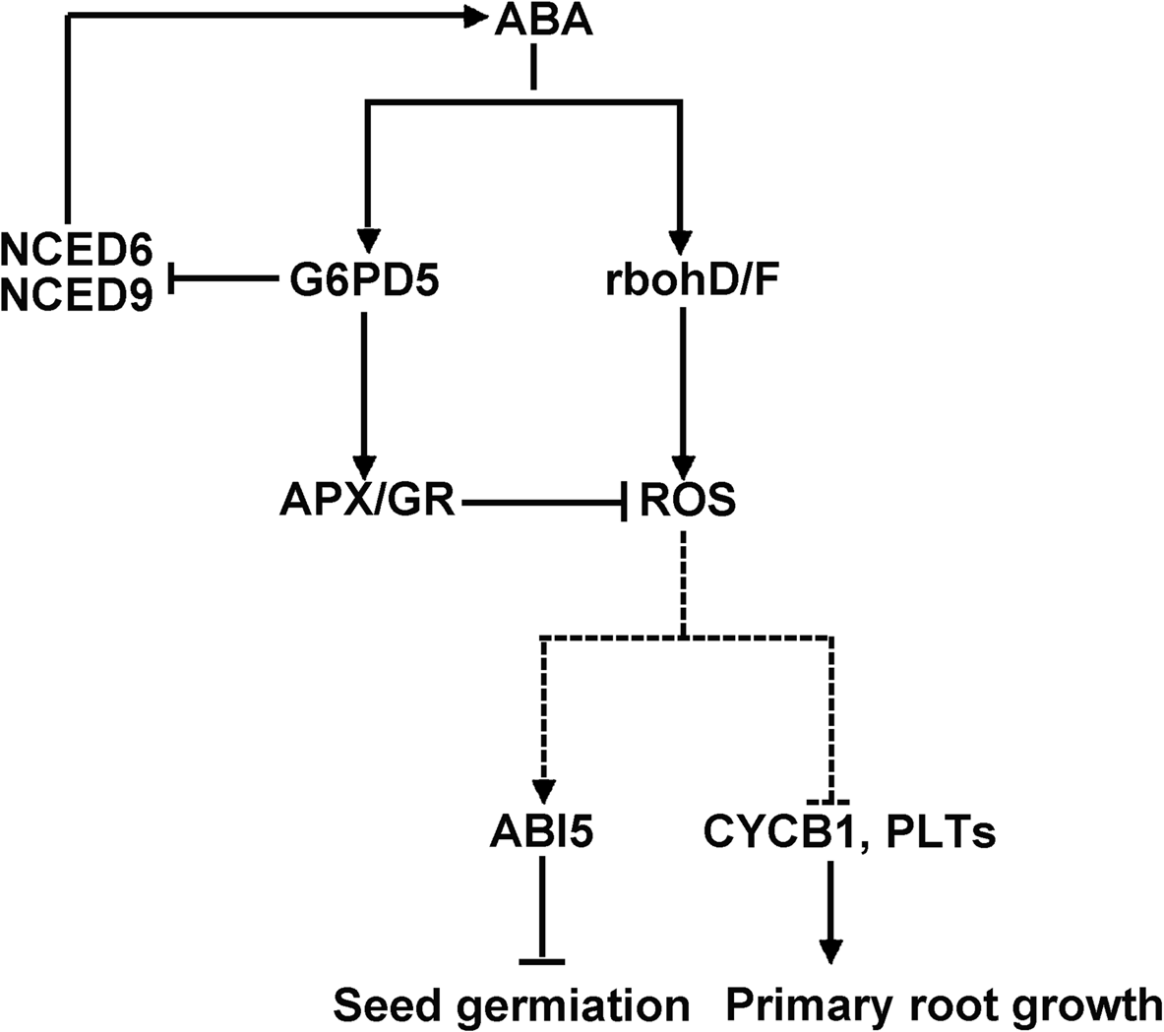
Schematic illustration of a proposed model during *Arabidopsis* seed germination and root growth. In this model, arrows indicate positive regulation and bars indicate negative regulation. Dotted arrows indicate results from the literature

Our results showed that ABA, H_2_O_2_, APX, GR and ABI are required for ABA-induced *G6PD5* gene function. Our findings point to a different node of this crosstalk that is activated by an increase in cytosolic G6PD5 and that is involved in dormancy and germination control. Based on these results, as well as those reported previously, we proposed a hypothetical model shown in Fig. 10. In this model, ABA induces *G6PD5*, which subsequently suppresses H_2_O_2_ generation by activating the NADPH oxidase in seeds and roots under ABA treatment. The enhanced *G6PD5* is involved in regulating key enzymes (APX and GR) in the ASC-GSH cycle by utilizing NADPH. The enhanced antioxidant ability can facilitate to maintain a steady-state level of H_2_O_2_ in cells, thus avoiding ROS damages to plant cells. G6PD5 is crucial for RAM maintenance in the presence of ABA by regulating the expression levels of *CYCB1*, *PLT1* and *PLT2.* Moreover, *G6PD5* affects genes in ABA biosynthesis and catabolism after ABA treatment and *G6PD5* probably acts upstream of *ABI* genes in the ABA signaling pathway.

## Additional file

Additional file 1: **Table S1.** Primer sequences used in the study. **Figure S1.** ROS levels in *g6pd5* and *OE* lines. 1-day-old seeds were grown vertically on 1/2 MS agar plates supplemented with 10 μM ABA for 6 h. Quantification of the H_2_DCF-DA fluorescence in *Arabidopsis* seeds with ABA treatment. **Figure S2.** H_2_O_2_ and O_2_^−^ levels in *g6pd5* and *OE* lines. 5-day-old seedlings were grown vertically on 1/2 MS agar plates supplemented with the 10 μM ABA for 6 h. **Table S2.** *Cis-acting* regulatory elements identified in the promoter region of *G6PD5* and *G6PD6*. The online search tool PlantCARE was used to detect putative *cis-acting* regulatory elements. (http://bioinformatics.psb.ugent.be/webtools/plantcare/html/). (DOC 610 kb)

## Abbreviations

ABA: Abscisic acid
ABI: Abscisic acid insensitive
APX: Ascorbate peroxidase
AsA: Ascorbate acid
Cy-G6PD: Cytosolic glucose-6-phosphate dehydrogenase
CYP707A: Aabscisic acid 8′-hydroxylase
DAB: 3, 3′-diaminobenzidine
G6PD: Glucose-6-phosphate dehydrogenase
GR: Glutathione reductase
GSH: Glutathione
H_2_DCF-DA: Dichlorodihydrofluorescein diacetate
H_2_O_2_: Hydrogen peroxide
NBT: Nitroblue tetrazolium
NCED: 9-cis-epoxycarotenoid dioxygenase
PPP: Pentose phosphate pathway
ROS: Reactive oxygen species
