# Supplementary material for: Involvement of G6PD5 in ABA response during seed germination and root growth in Arabidopsis
Source: BMC Plant Biol. 2019 Jan 30;19:44. doi: 10.1186/s12870-019-1647-8 (PMC6354342; doi:10.1186/s12870-019-1647-8)
Supplement: Supplementary file 1 — Table S1. Primer sequences used in the study. Figure S1. ROS levels in g6pd5 and OE lines. 1-day-old seeds were grown vertically on 1/2 MS agar plates supplemented with 10 μM ABA for 6 h. Quantification of the H2DCF-DA fluorescence in Arabidopsis seeds with ABA treatment. Figure S2. H2O2 and O2− levels in g6pd5 and OE lines. 5-day-old seedlings were grown vertically on 1/2 MS agar plates supplemented with the 10 μM ABA for 6 h. Table S2. Cis-acting regulatory elements identified in the promoter region of G6PD5 and G6PD6. The online search tool PlantCARE was used to detect putative cis-acting regulatory elements. (http://bioinformatics.psb.ugent.be/webtools/plantcare/html/). (DOC 610 kb) [file 12870_2019_1647_MOESM1_ESM.doc]

Supplementary Material

Table S1

| Gene name | Primer sequence 5’-3’ |
| --- | --- |
| *Actin2* | GTT GGG ATG AAC CAG AAG GA |
|  | CTT ACA ATT TCC CGC TCT GC |
| *qActin2* | TTTCCCGCTCTGCTGTTGT |
|  | TGTGCCAATCTACGAGGGTTT |
| *G6PD5* | CACCATGGGTTCTGGTCAATGGC |
|  | CAATGTAGGAGGGATCTAAATGTAG |
| *qG6PD5* | TCTTGCACTTCCTCCGTCTG |
|  | GCGTTCGTAAGCCTCTGG |
| *qG6PD6* | GTTGGTCCTCCGGTTTGC |
|  | CTTTCGGTCCTCGGCTTC |
| *qAtrbohD* | TGGAAGGATGGACTGGCATT |
|  | CTTGAGGAAGTTAGGTAAGTTAAGC |
| *qAtrbohF* | GACTTCTCAGAGCCGACGAA |
|  | CAATGCCAAGACCAACTAATAAGAG |
| *qAPX1* | GTGTTTTTGGTTGGGGGCTG |
|  | GTCTAAGCAGCAAAAGCGCA |
| *qGR2* | GGTCGCAAGCCCAACACAAAG |
|  | ACAGCCCAGATGGATGGAACAG |
| *qNCED6* | TGAGAGACGAAGAGAAAGAC |
|  | GTTCCTTCAACTGATTCTCG |
| *qNCED9* | AACCGCCGCTATGGTTTTAGACG |
|  | CCAGTCACCGGAAGGTTATGCAC |
| *qCYP707A3* | CTCTGTTTCTCTGTTTACTCCGATTTA |
|  | TGCAGCAAAACAGAGAAGATACG |
| *qCYP707A4* | GAAAGGAATACAGTACAGTC |
|  | GGATTAGATTTGGCTAACTAC |
| *qABI3* | GCTGCTGTGTTTTTGGAGTG |
|  | AGTCTTCTTGCCGCTGATTC |
| *qABI4* | GCTTCCCAACATCAACACAACC |
|  | TTGAGCGGAGGAAGTTGATGAG |
| *qABI5* | GAGAATGCGCAGCTAAAACA |
|  | GTGGACAACTCGGGTTCCTC |
| *qCYCB1;1* | TTGGCTGAGTTAGGCGTAATGC |
|  | GTGATGCTTGAGAGTGCTGGTC |
| *qPLT1* | TCGCCGGAAACAAAGAC |
|  | CCGATGGGAAGAGTGCTAC |
| *qPLT2* | ACTCTTCTTTGCCGCCTCACAT |
|  | CCCCCGATTTGCTCACTCC |
| *LBb1* | GCGTGGACCGCTTGCTGCAACT |
| *pGWB2* | ATTTGGAGACACGGGG |


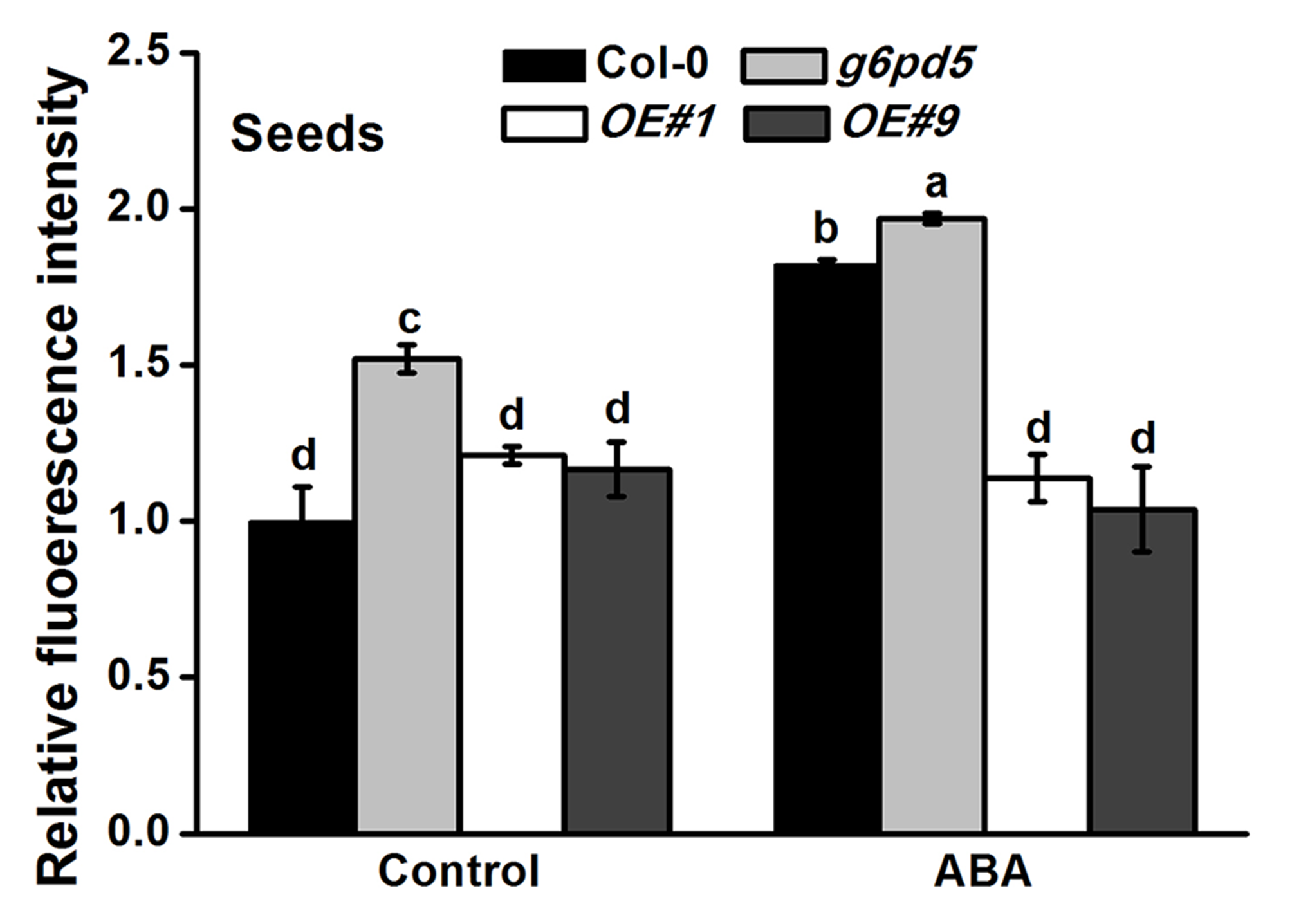
 Fig. S1


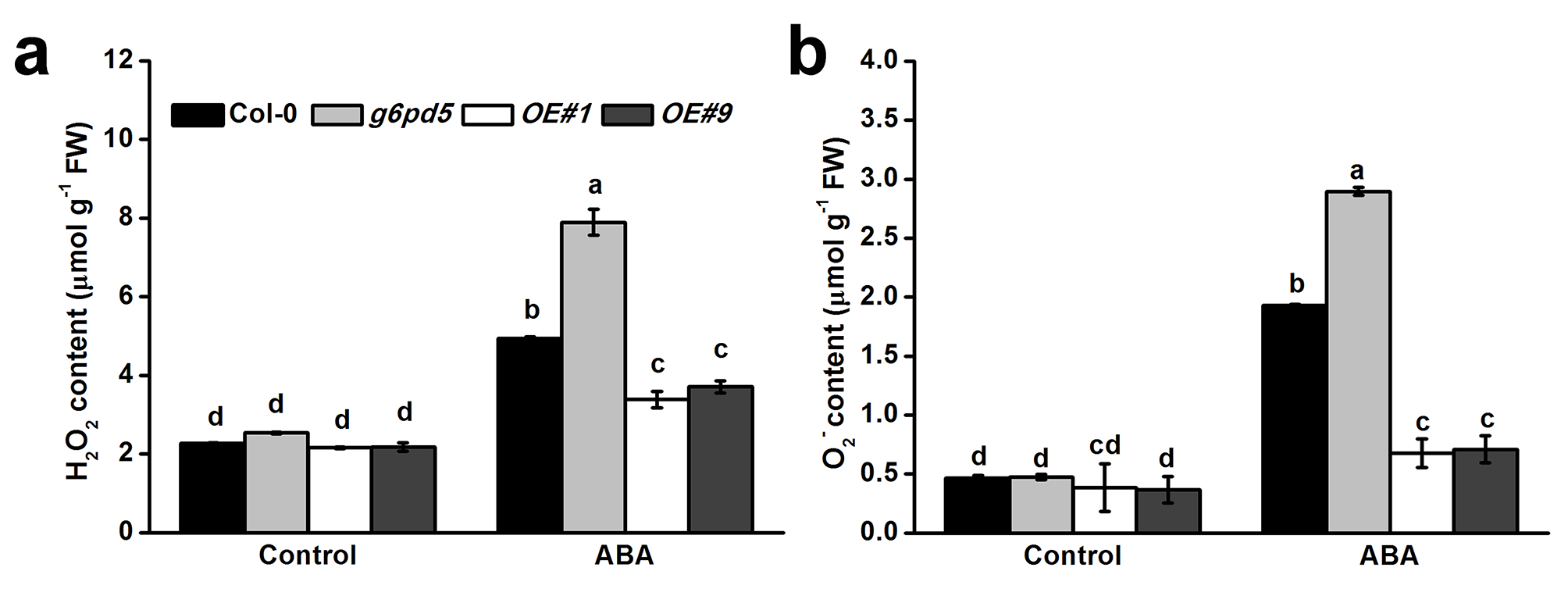


Fig. S2

Table S2

| *Cis-acting* regulatory elements identified in the promoter region of ***G6PD5***. |  |  |  |
| --- | --- | --- | --- |
| **Site name** | **Strand** | **Sequence** | **Function** |
| **ARE** | (-) | TGGTTT | *Cis-acting* regulatory element essential for anaerobic induction |
| **ABRE** | (-) | CACGTG | *Cis-acting* element involved in the abscisic acid responsiveness |
| **CGTCA-motif** | (+) | CGTCA | *Cis-acting* regulatory element involved in the MeJA-responsiveness |
| **HSE** | (-) | AAAAAATTTC | *Cis-acting* element involved in heat stress responsiveness |
| **MBS** | (-) | CAACTG | MYB binding site involved in drought-inducibility |
| **TATCCAT/C-motif** | (-) | TATCCAT | *Cis-acting* regulatory element; associated with G-box like motif; involved in sugar repression responsiveness |
|  |  |  |  |
|  |  |  |  |
|  |  |  |  |
| *Cis-acting* regulatory elements identified in the promoter region of ***G6PD6***. |  |  |  |
| **Site name** | **Strand** | **Sequence** | **Function** |
| **ARE** | (-) | TGGTTT | *Cis-acting* regulatory element essential for anaerobic induction |
| **CGTCA-motif** | (+) | CGTCA | *Cis-acting* regulatory element involved in the MeJA-responsiveness |
| **HSE** | (-) | AAAAAATTTC | *Cis-acting* element involved in heat stress responsiveness |
| **MBS** | (-) | CAACTG | MYB binding site involved in drought-inducibility |
| **TC-rich repeats** | (-) | ATTTTCTCCA | *Cis-acting* element involved in defense and stress responsiveness |
| **TGACG-motif** | (+) | TGACG | *Cis-acting* regulatory element involved in the MeJA-responsiveness |
